# Adversity, Coping Repertoire, and Psychological Well-Being of Chinese Older Immigrants in the United States

**DOI:** 10.1093/geroni/igaa057.2128

**Published:** 2020-12-16

**Authors:** Man Guo, Yi Wang, Jinyu Liu, Meredith Stensland, XinQi Dong

**Affiliations:** 1 University of Iowa, Iowa City, Iowa, United States; 2 Weatherhead East Asian institute, New York City, New York, United States; 3 The University of Texas at Austin, Austin, Texas, United States; 4 Rutgers University, New Brunswick, New Jersey, United States

## Abstract

Using data from 2,923 Chinese older immigrants in Chicago, this study aims to identify different patterns of coping repertoires of older immigrants, based on a combination of individual, family and community coping resources, and the optimal coping repertoire that is associated with the best psychological outcomes. The results of Latent Class Analysis revealed four types of coping repertoires: low-resource (43%), spouse-oriented (32%), community-oriented (15%), and multi-source coping repertoire (10%). Overall, immigrants who had multi-source coping repertoire reported the best psychological outcomes. However, the influence of coping repertoires varied based on specific adversities. Having community-oriented coping repertoire was more protective for widowed immigrants, whereas spouse- or community-oriented coping repertoire was more protective for those with poor health. For less-acculturated older immigrants, having community-oriented coping appears most beneficial to their well-being; and for older immigrants who perceived low filial support from their children, having multi-source coping was associated with better psychological well-being.

